# Salt-Tolerant PGPR Confer Salt Tolerance to Maize Through Enhanced Soil Biological Health, Enzymatic Activities, Nutrient Uptake and Antioxidant Defense

**DOI:** 10.3389/fmicb.2022.901865

**Published:** 2022-05-13

**Authors:** Muhammad Shabaan, Hafiz Naeem Asghar, Zahir Ahmad Zahir, Xiu Zhang, Muhammad Fahad Sardar, Hongna Li

**Affiliations:** ^1^Institute of Soil and Environmental Sciences, University of Agriculture, Faisalabad, Pakistan; ^2^Ningxia Key Laboratory for the Development and Application of Microbial Resources in Extreme Environments, North Minzu University, Yinchuan, China; ^3^Agricultural Clean Watershed Research Group, Institute of Environment and Sustainable Development in Agriculture, Chinese Academy of Agricultural Sciences, Beijing, China

**Keywords:** rhizobacteria, maize, salinity stress, enzymes, nutrient acquisition

## Abstract

Salt-tolerant plant growth-promoting rhizobacteria (PGPR) can improve soil enzyme activities, which are indicators of the biological health of the soil, and can overcome the nutritional imbalance in plants. A pot trial was executed to evaluate the effect of inoculation of different salt-tolerant PGPR strains in improving soil enzyme activities. Three different salinity levels (original, 5, and 10 dS m^–1^) were used and maize seeds were coated with the freshly prepared inocula of ten different PGPR strains. Among different strains, inoculation of SUA-14 (*Acinetobacter johnsonii*) caused a maximum increment in urease (1.58-fold), acid (1.38-fold), and alkaline phosphatase (3.04-fold) and dehydrogenase (72%) activities as compared to their respective uninoculated control. Acid phosphatase activities were found to be positively correlated with P contents in maize straw (*r* = 0.96) and grains (*r* = 0.94). Similarly, a positive correlation was found between alkaline phosphatase activities and P contents in straw (*r* = 0.77) and grains (*r* = 0.75). In addition, urease activities also exhibited positive correlation with N contents in maize straw (*r* = 0.92) and grains (*r* = 0.91). Moreover, inoculation of *Acinetobacter johnsonii* caused a significant decline in catalase (39%), superoxide dismutase (26%) activities, and malondialdehyde contents (27%). The PGPR inoculation improved the soil’s biological health and increased the uptake of essential nutrients and conferred salinity tolerance in maize. We conclude that the inoculation of salt-tolerant PGPR improves soil enzyme activities and soil biological health, overcomes nutritional imbalance, and thereby improves nutrient acquisition by the plant under salt stress.

## Introduction

Climate change has aggravated the harshness of environmental stressors and has badly affected agricultural crop productivity. At the same time, it is inevitable to increase agricultural production to meet the global food demand of a growing population, which is expected to reach 9 billion by 2050 (Rout, 2020). Excessive concentration of soluble salts in soil as well as water are responsible for lesser agricultural production and thereby, convert fertile lands to marginal lands leading to their abandonment (Ilangumaran and Smith, 2017). Soil salinization is considered one of the major constraints that not only limit agricultural output but also compromise soil fertility (Machado and Serralheiro, 2017). High salt concentrations usually occur in those regions that are characterized by high surface evaporation, low precipitation, and high temperature (Arora et al., 2018). In addition to that, continuous application of agrochemicals (fertilizers and pesticides) and various amendments with elevated salt concentrations, as well as the use of low-quality irrigation water are propagating salt accumulation to other cultivated areas (Enebe and Babalola, 2018). Toxic concentrations of soluble salts, predominantly NaCl, are present in the salt-affected areas, which induce osmotic stress, imbalance in nutrient uptake, and alterations in the plant metabolic system, thereby reducing plant growth (Deinlein et al., 2014). Moreover, excessive NaCl interferes with the soil microbial communities and their activities (Rath and Rousk, 2015) concomitant with detrimental impacts on organic matter degradation and nutrient cycling (Yan et al., 2015).

On a global scale, approximately 45 mha of farmland is estimated to be affected by salinity at an observed rate of 0.2–0.5 mha per year (CGIAR, 2016). To date, more than 20% of the agricultural land has been affected by elevated NaCl concentrations. If these conditions prevail at the same pace, agricultural land may lose 50% of its arability till 2050 (Din et al., 2019). Enormous salt concentrations in soil can result in hyperosmotic and hyperionic stresses that lead to a decline in plant growth, lessen nutrient uptake levels, and even result in plant death (Hidri et al., 2019).

Pakistan is an agriculture-based country that is estimated to have nearly 80% arid to semiarid areas. Soil salinization is one of the major limiting factors in Pakistan’s agriculture, and this problem has been further aggravated by the high evapotranspiration rate that prevails in the country with 8% humidity and 12% subhumid climatic conditions (Lal, 2018). Evapotranspiration rate is usually very high in June, i.e., 7.6 mm, and thereby, exceeding the precipitation rate (Hayat et al., 2020). In Pakistan, approximately 33% (6.8 mha) area of cultivation has been severely affected by salinity stress (Aslam et al., 2017). It not only disturbs the physicochemical properties of soil but also negatively affects the biological properties of soil.

Maize (*Zea mays* L.) ranks as the third most important cereal crop in Pakistan and it contributes to the value addition of agriculture and gross domestic product (GDP) by 2.1 and 0.4%, respectively (Abbas et al., 2017). Many studies have emphasized that global maize production must be doubled to meet the growing demands of living beings in the developing world (Meng et al., 2016). Maize is slightly sensitive to salinity stress, and at higher salinity levels, its production is severely affected, and salinity stress has become one of the major limiting factors regarding maize production (Farooq et al., 2015; Soothar et al., 2021). To achieve better maize growth and production, a suitable alternative must be sought to ensure sustained maize production even under excessive salt concentrations.

Different approaches are being used to manage salt-affected soils, such as the use of tube wells for irrigation, application of different chemicals such as gypsum, acids, and different organic amendments development of salt-tolerant cultivars, and inoculation of plant growth-promoting rhizobacteria (PGPR) strains to seeds. However, the use of physicochemical approaches is limited on account of their high cost and impracticability. The PGPR activities may alter the physiology of the plant’s root growth and support plants coping with salt stress (Azcón et al., 2013). Moreover, PGPR produces 1-aminocyclopropane-1-carboxylic acid (ACC) deaminase, which degrades ACC, a precursor of ethylene, which is very critical regarding salt tolerance in plants (Sarkar et al., 2019). PGPR can allocate its resources toward the production of different enzymes (dehydrogenases, phosphatases, and urease, etc.) that degrade organic matter followed by the mineralization of essential soil nutrients (Bezemer et al., 2010; Eisenhauer et al., 2010). These enzymes are believed to be linked to soil health and plant growth (Hidri et al., 2019; Sardar et al., 2021). These enzymes are responsible for increasing the availability of essential nutrients and, in this way, ensure nutrient availability for plant uptake. Overall, these soil enzymes may impose significant impacts on soil biology, growth, and nutrient uptake by plants and their management may be applied to stressed environments. Various studies have reported the role of microbes in association with plants in conferring salinity tolerance in plants (Hidri et al., 2016; Vimal et al., 2017; Vimal and Singh, 2019).

On this basis, this study was planned with the following objectives: (i) to compare the potential of salt-tolerant PGPR in improving soil enzyme activities under salt stress and (ii) to evaluate the possible role of soil enzymes in alleviating the nutritional imbalance in maize and improving its growth. We hypothesized that salt-tolerant rhizobacterial strains that possessed the potential to not only exhibit plant growth-promoting traits but also improve soil enzyme activities under varying levels of salt stress may improve the soil health and its ability to regulate the supply of essential nutrients for plant uptake.

## Materials and Methods

Rhizobacteria were previously isolated from maize that was grown in the salt-affected areas and evaluated for salt tolerance, plant growth-promoting traits, and extracellular enzyme production (Shabaan et al., 2021). Based on the results, ten strains were selected and were further investigated in a pot trial to test their efficacy in conferring salt tolerance to maize. Improvement in the soil enzyme activities after inoculation and a resultant increase in the plant uptake of essential nutrients were also correlated with plant growth and yield parameters. PGPR attributes of the rhizobacterial isolates are given in Table 1.

**TABLE 1:** Results of plant growth-promoting traits of rhizobacterial strains used in pot trial.

Strains	Gram staining (gram)	EPS	ACC deaminase	Siderophore production	Phosphate solubilization	Catalase	Oxidase	Urease	IAA activity	Phosphatase		DH
										Acid	Alkaline	
SHM-2	-	+	+	+	+	+	+	+	71.99	86.59	94.52	9.59 ± 0.10
SHM-5	+	++	++	+ +	++	+	+	+	185.49	158.72	270.02	10.44 ± 0.12
SHM-7	+	++	++	+	++	+	+	+	197.78	164.78	356.93	10.59 ± 0.17
SHM-11	-	+	+	+	+	+	+	+	108.89	102.81	141.74	9.24 ± 0.28
SHM-12	-	+	+	+	+	+	+	+	147.66	103.72	137.91	9.48 ± 0.38
SHM-13	+	++	++	+	++	+	+	+	152.85	160.35	248.15	10.95 ± 0.35
SHM-18	-	+	+	+	+	+	+	+	73.77	92.65	109.67	9.13 ± 0.28
SUA-14	-	+ +	++	++	+ +	+	+	+	258.90	167.83	373.91	11.98 ± 0.38
S1-3	-	+ +	+	+	+	+	+	+	118.07	105.00	148.57	10.61 ± 0.07
S3-26	-	+ +	+	+ +	+	+	+	+	80.24	139.00	185.39	8.88 ± 0.41

*DH **=** dehydrogenase activities (mg TPF/mg cell dry wt h^–1^), ALP = alkaline phosphatase activities (U mL^–1^), ACP = acid phosphatase activities (U mL^–1^), IAA = indole acetic acid activities (μg mL^–1^), ACC deaminase = 1 amino cyclopropane-1-carboylic acid activities, EPS = exopolysaccharide production (Shabaan et al., 2021).*

Details of different procedures during the experimental trial are given below.

### Inoculum Preparation

For the preparation of fresh inoculum of each of the selected rhizobacterial strains, Lauria Bertini (LB) broth media was used with the following recipe (10 g l^–1^) NaCl: 10, tryptone: 10, and yeast extract: 5. A pure single colony of each selected strain was inoculated to LB broth and was incubated at 28 ± 2°C for 48 h to prepare bacterial culture. Bacterial cells were harvested by centrifugation at 4,000 *g* and 4°C for 15 min. The pellet obtained was resuspended in the sterilized distilled water. The optical density of the bacterial culture was maintained at 10^7^–10^8^ colony-forming unit (CFU) ml^–1^ by using a UV-visible spectrophotometer (Mortensen et al., 2021).

### Seed Inoculation

Spring maize cultivar (31P41) was purchased from an authorized seed company and the inocula of rhizobacterial strain were carefully coated on the seeds. Before inoculation, seeds were washed with distilled water followed by their treatment with 2% sodium hypochlorite solution for 1–2 min. Then, after washing, the seeds were dipped in 95% ethanol for 30 s. Peat-based slurry and sugar solution (10%) were used for the seed inoculation (inoculum to peat ratio: 1:1), whereas for control treatments, seeds were coated with sterilized broth.

### Pot Trial

The experiment was designed to explore the effect of rhizobacterial inoculation on soil enzyme activities and antioxidants production. Calculated quantities of NaCl were added to the soil to develop electrical conductivity (EC) levels of 5 and 10 dS m^–1^, 2 weeks before the trial to ensure homogeneous mixing of salt in the soil. Experimental units were arranged according to a completely randomized design (CRD) under the factorial arrangement. Six seeds of maize were sown in each pot and three seedlings per pot were maintained after germination. Moreover, before pot filling, each pot was lined with polythene sheets to avoid leaching losses. A total of 10 kg of uniformly mixed and air-dried soil was filled in each pot having EC 1.58 dS m^–1^, pH 7.7, organic matter (OM) 0.61%, saturation percentage 36.98%, available phosphorus 6.9 mg kg^–1^, extractable potassium 165.7 mg kg^–1^, and cation exchange capacity (CEC) 1.49 C molc kg^–1^. The experiment was performed for a period of 110 days, and upon harvesting, growth and yield parameters were measured by following suitable protocols.

### Parameters Studied

#### Chlorophyll Contents and Relative Water Contents

The soil plant analysis development (SPAD) value was measured by using a chlorophyll meter (SPAD-502) at 10:00 a.m., whereas chlorophyll a, chlorophyll b, and carotenoid contents were measured by following the method of Armon (1949). Acetone extract (80% v/v) was used for determining the chlorophyll and carotenoids contents through spectrophotometer at 663, 645, and 480 nm wavelengths for chlorophyll a, chlorophyll b, and carotenoids, respectively, and were expressed as mg g^–1^ fresh weight.

Chl “a” = [12.7 (OD 663) – 2.69 (OD 645) × $\frac{\mathrm{Volume}\mathrm{of}\mathrm{sample}}{1000}$ × Weight of fresh tissue]

Chl “b” = [22.9 (OD 645) – 4.68 (OD 663) × $\frac{\mathrm{Volume}\mathrm{of}\mathrm{sample}}{1000}$ × Weight of fresh tissue]

Carotenoids = $\frac{\text{Acar}}{\text{Em}}$ × 100,

where

Em = 2,500 and A*^car^* = OD 480 + 0.114 (OD 663) – 0.638 (OD 645)

For the determination of relative water contents, the method of Barrs and Weatherley (1962) was followed;


(1) $$\begin{array}{c} \mathrm{Relative}\mathrm{water}\mathrm{contents}\left(\mathrm{RWCs}\right)=\\ \frac{\mathrm{Fresh}\mathrm{weight}-\mathrm{Dry}\mathrm{weight}}{\mathrm{Fully}\mathrm{turgid}\mathrm{weight}-\mathrm{Dry}\mathrm{weight}}\times 100 \end{array}$$

To get a turgid weight of the leaves, they were placed in distilled water for 18 h in the dark at room temperature. Leaves were blotted carefully with tissue paper for the determination of turgid weight, while dry weight was obtained after drying the leaves in an oven grain fresh weight (g) at 70°C for 72 h.

#### Growth and Yield Parameters

Upon harvesting, data regarding maize growth parameters and yield parameters were measured.

#### Nutrient Analysis in Plant

Determination of N, P, K, and Na contents in plant matter and grains after grinding and digestion is as follows.

##### Digestion

Plant samples were digested by following the method of Wolf (1982) where ground plant samples (0.5 g) were used. Completion of the digestion process was indicated by the appearance of colorless liquid in the flask. Later, the material was diluted with distilled water followed by its filtration into a 50-ml volumetric flask, and distilled water was used again to fill the flask up to the mark. The digested material was stored for N, P, and K analysis.

##### Nitrogen, Phosphorus, and Potassium Determination

For the determination of N, P, K, and Na contents in different plant parts, Kjeldahl ammonia distillation apparatus, spectrophotometer (ANA-720W, Tokyo Photoelectric Company Limited, Japan), and Flame photometer (Jenway PFP-7) were used.

#### Antioxidant Production

##### Enzyme Extract

Enzyme extraction was carried out by the homogenization of maize leaves in phosphate buffer (7.0) in prechilled pestle and mortar by following the method used by Pandey et al. (2003). The extract was centrifuged in a cooled centrifuge at 9,000 *g* for 20 min. The supernatant was analyzed for catalase (CAT), superoxide dismutase (SOD) activities, and malondialdehyde (MDA) contents by following suitable protocols.

##### Catalase [mM Hydrogen Peroxide Mint^–1^ g^–1^ Fresh Weight]

Catalase (CAT) activities in enzyme extract were determined by following the method of Hugo and Lester (1984), where decomposition of hydrogen peroxide (H_2_O_2_) was observed spectrophotometrically at 240 nm. Then, 3 ml of reaction mixture comprised 2 ml of enzyme extract and 10 mM H_2_O_2_.

##### Superoxide Dismutase (Nmol Mint^–1^ mg^–1^ Protein)

Total SOD activity in the crude enzyme extract was assayed by using the modified nitroblue tetrazolium (NBT) substrate method. The reaction mixture (2 ml) comprised of phosphate buffer (50 mM; pH 7.8) having ethylenediaminetetraacetic acid (EDTA) (2 mM), L-methionine (9.9 mM), NBT (55 μM), and 0.025% Triton X-100, and the reaction was started by the illumination of the sample under a fluorescent lamp and terminated by covering the test tubes with aluminum foil. SOD activity was measured by calculations given by Beauchamp and Fridovich (1971).

##### Lipid Peroxidation (Malondialdehyde Contents)

Levels of lipid peroxidation in maize leaves were determined by Sheng and Zhu (2019) and comprised of homogenization of leaves in the presence of 10% trichloroacetic acid (TCA) followed by their homogenization at 9,000 g for 20 min. A reaction mixture consisting of aliquot (2 ml), 0.6% thiobarbituric acid (2 ml) in 10% TCA was heated for 15 min at 100°C followed by its quick cooling in an ice bath. The mixture was centrifuged at 9,000 *g* for 20 min and absorbance of the resultant supernatant was measured at 532 and 450 nm. MDA contents were expressed as nmol mint^–1^ mg^–1^ protein.

##### Proline Determination (μMole G^–1^ Fresh Weight)

Proline determination in the samples was done according to the method of Bates et al. (1973) where a 0.5-g plant sample was homogenized in sulfosalicylic acid followed by its filtration. The filtrate was mixed with acid ninhydrin (2 ml) and glacial acetic acid (2 ml) and the reaction terminated in an ice bath. Toluene was used for the extraction of the reaction mixture. The absorbance of the reaction mixture was determined at 520 nm. Proline concentration was determined using a standard curve.

#### Soil Enzyme Activities

For the measurement of soil enzyme activities, rhizosphere soil that was closely attached to plant roots was taken in the laboratory and stored in a refrigerator at -4°C in polythene bags.

##### Soil Urease Activity

Soil urease activity was determined by following the method of Tabatabai (1972) and was based on the determination of ammonium released during the steam distillation of soil suspension with 0.2 g MgO with boric acid (H_3_BO_3_) as a receiver solution when the soil was incubated with tris(hydroxymethyl)aminomethane (THAM) buffer, urea solution (analytical grade), and toluene at 37°C for 2 h. It was expressed as μg NH_4_^+^ released per gram of soil per 2 h.

##### Soil Phosphatase Activity

Activities of acid and alkaline phosphatase in the soil samples were determined by following the method used by Tabatabai and Bremner (1969) and were based on the colorimetric estimation of *p*-nitrophenol released by the incubation of soil with *p*-nitrophenyl phosphate solution and toluene. Two different types of modified universal buffer (pH 6.5 for acidic) and (pH 11 for alkaline) phosphatase were prepared. The intensity of the yellow color was spectrophotometrically observed at 410 nm.

##### Soil Dehydrogenase Activity

Soil dehydrogenase activities were measured based on the colorimetric determination of 1,3,5-triphenylformazan (TPF) formed on account of microbially mediated reduction of 2,3,5-triphenyltetrazolium chloride (TTC), which was devised by Lenhard (1956). The intensity of reddish color developed by the reduction of TPF was measured at 485 nm with methanol as a blank. Based on the values of absorbance of standard TPF solution in methanol, a calibration curve was drawn to quantify the dehydrogenase activity and was expressed as μg TPF formed per h per g dry weight of soil.

### Microbial Identification

Bacterial strains were identified using 16S rRNA sequencing. Identification was done by comparing the partial sequences of the isolated strains with those existing in the GenBank database. Universal primer sets, forward primer 27F 5’ (AGA GTT TGA TCM TGG CTC AG) 3’ and reverse primer 1492R 5’ (TAC GGY TAC CTT GTT ACG ACT T) 3’, were used for this purpose.

### Statistical Analysis

The data of each parameter were separately analyzed, and all the statistical analyses were performed using software IBM SPSS v19.0. The experiment consisted of thirty-three treatments, each with three replications. Means, SEs, and residues were calculated using Microsoft Excel software (version 2019, United States). Tukey’s honestly significant difference (HSD) test was used as a *post hoc* test to conduct pairwise comparisons between different treatments at *p* ≤ *0.05*. Moreover, the Pearson correlation between soil enzyme activities and different growth-related parameters was performed in RStudio, and a correlogram was constructed using the built-in “cor” function and the publicly available package “corrplot,” as per R-project instructions (RStudio Team, 2019).

## Results

Salinity stress has a damaging effect on soil enzyme activities as well as growth, yield, and physiological attributes of maize, and these effects were more pronounced at higher salinity levels. However, rhizobacterial inoculation caused a considerable improvement in all the measured plant traits at all the salinity levels. Different rhizobacterial strains responded differently toward applied salinity stress and regulated plant growth as compared to the uninoculated contaminated control.

### Growth and Yield Attributes

**FIGURE 1:**
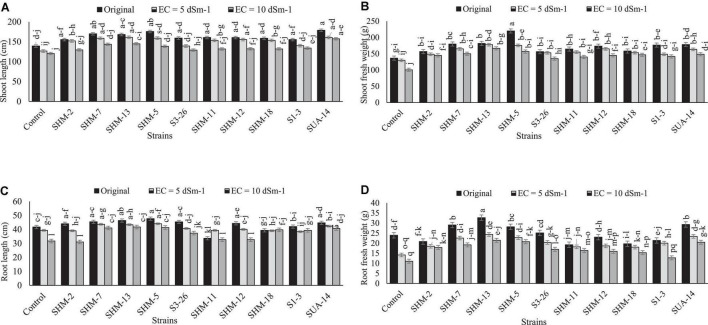
Effect of rhizobacterial inoculation on growth parameters, i.e., **(A)** shoot length (cm), **(B)** shoot fresh weight (g), **(C)** root length (cm), and **(D)** root fresh weight (g) of maize under salt-stressed pot conditions. Bars sharing similar letters are statistically non-significant at *p* ≤0.05.

**FIGURE 2:**
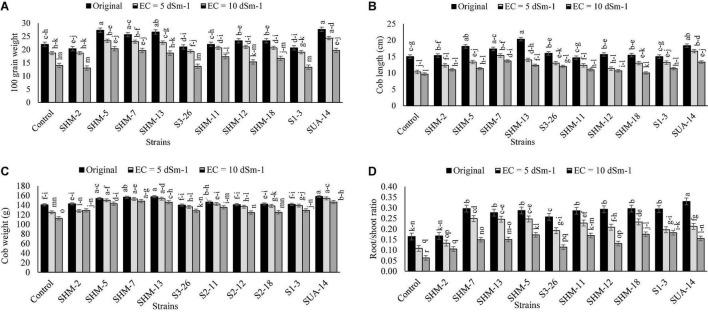
Effect of rhizobacterial inoculation on yield parameters, i.e., **(A)** 100-grain weight (g), **(B)** Cob length (cm), **(C)** Cob weight (g), and **(D)** root/shoot ratio of maize under salt-stressed pot conditions. Bars sharing the same letters are statistically non-significant at *p*≤0.05.

Results indicated that salinity stress decreased the overall growth and yield attributes, where the highest salinity stress (10 dS m^–1^) exhibited more adverse impacts as compared to the lower levels as well as control as it decreased the shoot length (1.2-fold), shoot fresh biomass (26%), root length (9%), root fresh weight (11%), root/shoot ratio (5%), 100-grain weight (36%), and cob length (35%) in the absence of inoculation and comparison with the control treatment (Figure 1). However, among all the inoculated rhizobacterial strains, maximum increment in the shoot length (31%) was found by the inoculation of SUA-14, whereas inoculation of SHM-13 caused a significant promotion in the shoot fresh biomass (66%), root length (20%), and root fresh weight (94%) in comparison with their respective uninoculated control at 10 dS m^–1^ salinity level. Similarly, results regarding yield parameters (Figure 2) exhibited that, at the highest salinity level (10 dS m^–1^), maximum increment in the cob length (41%) and cob weight (32%) was observed with SHM-13 inoculation, followed by SUA-14, which increased cob length and cob weight by 38 and 30%, respectively. Similarly, a maximum increment in the 100-grain weight was found due to inoculation with SHM-7 (45%).

### Physiological Attributes

**FIGURE 3:**
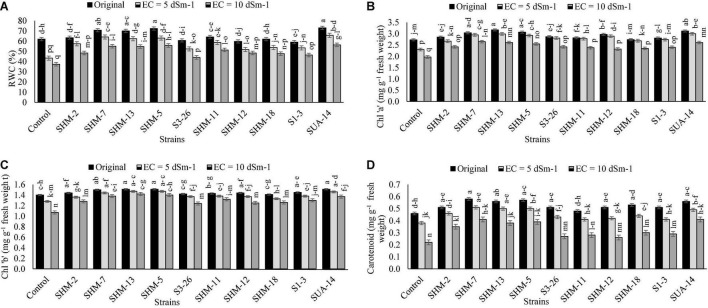
Effect of rhizobacterial inoculation on the physiological attributes, i.e., **(A)** Relative water contents (%), **(B)** Chlorophyll “a”. Effect of rhizobacterial inoculation on relative water contents (RWCs) (%), **(C)** Chlorophyll “b” (mg g^–1^ fresh weight), and **(D)** carotenoid (mg g^–1^ fresh weight) contents of maize under salt-stressed pot conditions. Bars sharing the same letters are statistically non-significant at *p*≤0.05.

Results regarding the physiological attributes (relative water contents, chl “a” and “b,” and carotenoids contents) (Figure 3) revealed that, in the absence of bacterial inoculation, maximum adverse impacts were observed at a higher salinity level of 10 dS m^–1^ as it decreased the SPAD (23%), chl “a” (28%), chl “b” (24%), and carotenoid (52%) contents as compared to the control treatment. However, a maximum increment in the relative water contents (52%) was found with inoculation of SUA-14 followed by SHM-5 (50%) as compared to their respective uninoculated control. Similarly, the maximum increase in the chlorophyll “a” (35%) and chlorophyll “b” (33%) contents was found due to inoculation with SHM-7. Moreover, SHM-7 caused a significant uplift in the carotenoid (88%) contents followed by SUA-14 (85%).

### Antioxidant Production

**FIGURE 4:**
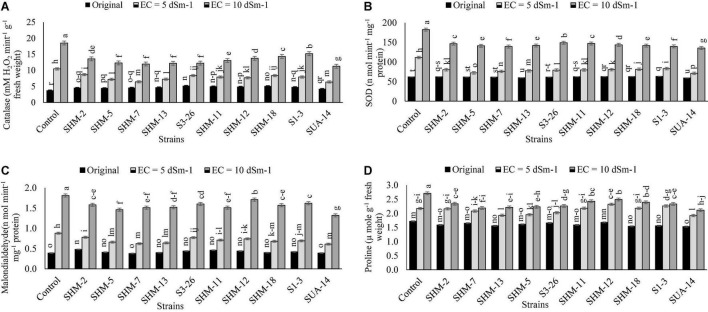
Effect of rhizobacterial inoculation on the antioxidants, i.e., **(A)** catalase [mM hydrogen peroxide (H_2_O_2_) mint^–1^ g^–1^ fresh weight], **(B)** superoxide dismutase (SOD) (n mol mint^–1^ mg^–1^ protein), **(C)** malondialdehyde (n mol mint^–1^ mg^–1^ protein), and **(D)** proline (μ mole g^–1^ fresh weight) activities of maize under salt-stressed pot conditions. Bars sharing the same letters are statistically non-significant at *p*≤0.05.

Results of antioxidant production (Figure 4) revealed that salinity stress considerably increased the antioxidant activities in the uninoculated treatments where greater antioxidant activities, i.e., CAT (3.8-fold), SOD (1.95-fold), and malondialdehyde (3.9-fold), were observed at 10 dS m^–1^ salinity level than the control treatment. Similarly, among all the inoculated strains, a maximum decline in the CAT (39%), SOD (26%), proline (22%) activities, and malondialdehyde (27%) contents were found due to inoculation with SUA-14 at a salinity level of 10 dS m^–1^. Similarly, SHM-13 inoculation also reduced CAT and SOD activities by 35 and 24%, respectively. Among other strains, inoculation of SHM-7 caused a significant reduction in the malondialdehyde contents by 20%, respectively, over its corresponding control.

### Soil Enzyme Activities

**FIGURE 5:**
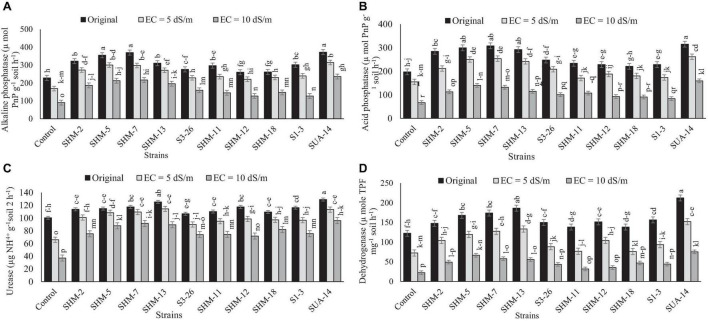
Effect of rhizobacterial inoculation on the soil enzyme activities, i.e., **(A)** alkaline phosphatase (μ mol PnP g^–1^ soil h^–1^), **(B)** acid phosphatase (μ mol PnP g^–1^ soil h^–1^), **(C)** urease (μg NH_4_^+^ g^–1^soil 2 h^–1^), and **(D)** dehydrogenase (μ mole TPF mg^–1^ soil h^–1^) under salt-stressed pot conditions. Bars sharing the same letters are statistically non-significant at *p*≤0.05.

In the same way, salinity stress significantly decreased the soil enzyme activities under salt stress and among all the salinity levels and control, the maximum decline in the soil enzyme activities, i.e., urease (63%), dehydrogenase (82%), alkaline (61%), and acid phosphatase (66%), was observed at higher salinity level (10 dS m^–1^) in comparison with the control (Figure 5). However, rhizobacterial inoculation improved the soil enzyme activities where different strains responded differently. Maximum increment in the acid phosphatase (1.38-fold), alkaline phosphatase (3.04-fold), urease (1.58-fold), and dehydrogenase (72%) activities was observed due to inoculation of SUA-14 followed by SHM-13, which was also efficient in improving alkaline phosphatase and urease activities by 2.8- and 1.4-fold, respectively. In addition, inoculation of SHM-7 improved soil acid phosphatase activities by 1.1-fold.

### Nutrient Analysis

**FIGURE 6:**
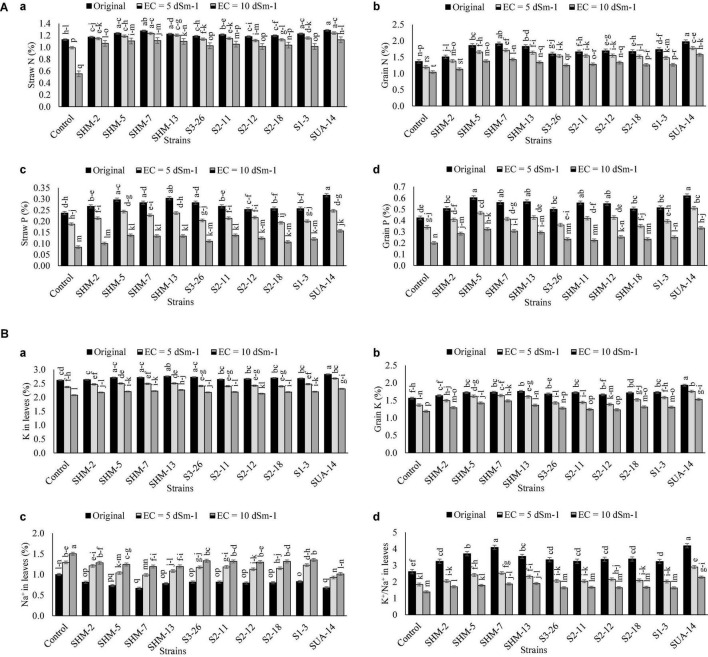
**(A)** Effect of rhizobacterial inoculation on the nutrient uptake in different plant parts. (a) Straw N (%), (b) Grains N (%), (c) Straw P (%), and (d) Grains P (%) of maize under salt-stressed pot conditions. Bars sharing the same letters are statistically non-significant at *p*≤0.05. **(B)** Effect of rhizobacterial inoculation on the nutrient uptake in different plant parts. (a) K in leaves (%), (b) Grains K (%), (c) Na in leaves (%) of maize, and (d) K^+^/Na^+^ ratio in maize leaves under salt-stressed pot conditions. Bars sharing the same letters are statistically non-significant at *p*≤0.05.

Results of nutrient (NPK) analysis in the aboveground plant parts are given in Figures 6A,B, which indicated that SUA-14 exhibited significant outcomes in terms of essential nutrients uptake in the aboveground plant parts as its inoculation increased straw N contents (1.04-fold), grain N contents (47%), straw P contents (92%), grain P contents (65%), leaves P contents (11%), and grain P contents (29%) at the salinity level of 10 dS m^–1^. Similarly, SUA-14 caused a maximum reduction in the Na contents of maize leaves (33%) with a simultaneous improvement in the K^+^/Na^+^ ratio by 54% over its uninoculated control.

### Correlation Analysis

**FIGURE 7:**
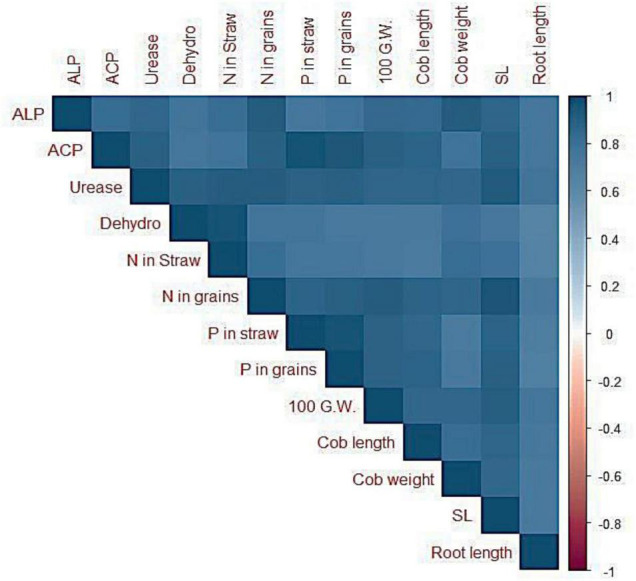
Correlation graphs between soil enzyme activities and growth and yield parameters as well as nutrient acquisition. ACP = Acid phosphatase; ALP = Alkaline phosphatase; DH = Dehydrogenase; CL = cob length; CW = Cob weight; SL = Shoot length; RL = Root length; 100 G.W. = 100 grain weight.

The Pearson correlation depicted existence of a strong correlation between soil enzyme activities, plant nutritional acquisition, and maize growth attributes (Figure 7). Alkaline phosphatase activities were positively correlated to grain P contents (*r* = 0.77), cob length (*r* = 0.83), cob weight (*r* = 0.92), shoot length (*r* = 0.87), and root length (*r* = 0.89). In addition, acid phosphatase activities exhibited strong positive correlation with grain P contents (*r* = 0.94), cob length (*r* = 0.88), cob weight (*r* = 0.78), shoot length (*r* = 0.90), and root length (*r* = 0.74).

### Bacterial Identification

Four most successful rhizobacterial strains were identified by 16S rRNA sequencing. Based on the results, SUA-14, SHM-13, SHM-5, and SHM-7 were identified as *Acinetobacter johnsonii* (MW681519), *Bacillus altitudinis* (MW681518), *Bacillus subtilis* (MW681516), and *Bacillus altitudinis* (MW681517), respectively.

## Discussion

Salinity stress has been globally reported to affect plant growth via different ways, such as via induction of osmotic stress, nutritional imbalance, ionic stress, and production of reactive oxygen species. Increased salt concentrations not only affect the physicochemical properties of the soil but also interfere with microbial activities such as microbial respiration and soil enzyme activities. Microbially produced extracellular enzymes are released into the soil, which facilitate the mineralization of organic matter in the soil and release essential plant nutrients and thereby improve the soil fertility status and biological health. This study revolved around the inoculation of salt-tolerant rhizobacteria to improve the soil enzyme activities as well as soil biological health and, in this way, to confer salinity tolerance in maize under salt stress. Results of this study revealed that salinity stress caused a negative impact on the soil enzyme activities, growth, yield, and physiological attributes of the maize plant. However, the application of rhizobacterial strains isolated from maize rhizosphere overcomes the drastic effects of salinity. PGPR-mediated improvement in the growth, yield, and physiological attributes of maize might be due to different mechanisms such as ACC deaminase production (Afridi et al., 2019), exopolysaccharides production (Atouei et al., 2019), phosphate solubilization (Kadmiri et al., 2018), indole-3-acetic acid production (Zahir et al., 2010; Myo et al., 2019), and siderophore production (Kumar et al., 2018).

### Growth and Yield Attributes

Salinity-mediated reduction in the plant’s growth attributes has been reported (Ali et al., 2017; Tian et al., 2020). Moreover, the decline in the yield of maize under salinity stress is also reported (Yasin et al., 2018a; Azadikhah et al., 2019). Under salinity stress, plants experience an osmotic effect and specific ion toxicity, which, ultimately, led to a significant decrease in plant growth. Therefore, the reduction in plant growth and yield traits in this experiment might be due to salt accumulation in the aboveground plant parts. Rhizobacterial inoculation improved the growth as well as yield of maize under salinity stress in comparison with their uninoculated unstressed control. These results are in line with the previous reports (Hussein and Joo, 2018; Upadhyay et al., 2019; Javed et al., 2020). The increase in plant growth might be correlated with the production of auxin by inoculated rhizobacterial strains that may have enhanced the cell division as well as elongation in the seedlings (Kadmiri et al., 2018). Moreover, improved essential nutrient uptake concomitant with a decline in the uptake of Na^+^ might be another reason for the increased plant growth under inoculated treatments (Hidri et al., 2019). This might be due to microbially mediated soil enzyme activities, which improved the soil properties and, hence, its ability to provide essential nutrients under salinity stress.

### Physiological Attributes

Salinity stress was found to be negatively correlated to chlorophyll contents (chlorophyll “a” and “b” and carotenoid contents). Similar results have already been reported by previous studies (Akladious and Mohamed, 2017; Latef et al., 2020; Tiwari et al., 2020). A decline in the chlorophyll contents under salinity stress might be due to the induction of osmotic stress that may have interfered with the uptake of essential minerals (Ali et al., 2017) or due to specific uptake followed by accumulation of Na^+^ in the leaves that might have hampered chlorophyll and ultimately led to its reduction (Narimanim et al., 2020). In addition, this decline may be due to oxidative stress that might have damaged the chloroplast membrane. Inoculation with rhizobacterial strains uplifted chlorophyll “a” and “b” and carotenoid contents as compared to control, and each rhizobacterial strain responded differently. Assessment of leaf water content is an efficient tool to describe the water status of the plant and a decline in the relative water content (RWC) is the first notable effect of salt stress (Win et al., 2015). In this scenario, salinity stress was shown to negatively affect RWC in maize (Fardus et al., 2017; Yasin et al., 2018b; Shahverdi et al., 2020). Under the impact of salinity, plants experience osmotic stress, which leads to reduced water uptake. Moreover, excessive production of abscisic acid (ABA) under salinity stress induces stomatal closure that leads to low or no water uptake by the roots entailing low relative water contents of the plants (Polash et al., 2018). Microbially mediated improvement in the relative water contents (RWCs) of plants has already been documented (Ji et al., 2020; Li et al., 2020). Increment in the RWC under inoculated treatments might be due to microbially induced enhancement in the root length and diameter that may have helped the plant to uptake more water under the exact salinity situation (Ali et al., 2017).

### Nutritional Attributes

Our results also depicted that the uptake of essential nutrients (NPK) was reduced under salinity stress (Ansari et al., 2019; Bistgani et al., 2019; Chrysargyris et al., 2019). This decline in the nutrient uptake might be due to salinity-induced water homeostasis and mismanagement in the ionic allocation within plant tissues. Moreover, high contents of Na^+^ under salinity stress exhibited antagonistic behavior with essential nutrients, particularly K^+^ and, hence, caused a significant reduction in its uptake. The current study demonstrated that inoculation of salt-tolerant rhizobacteria improved straw and grain nutrient content and caused a significant decline in the Na^+^ contents of plant with a simultaneous increase in the K^+^/Na^+^ ratio. One possible reason might be the production of exopolysaccharides by rhizobacteria, which might have reduced the availability of Na^+^ to plant roots, leading to an incline in the K^+^/Na^+^ ratio (Ansari et al., 2019; Vimal and Singh, 2019). The effect of bacterial inoculation in alleviating the salinity-mediated nutritional imbalance in plants has been reported (Yasmeen et al., 2019; Azarmi-Atajan and Sayyari-Zohan, 2020).

### Antioxidant Production

Moreover, we observed an increased production of different antioxidants (CAT, SOD, MDA, and proline contents) under salt-stressed conditions in this experiment, which are in line with the findings of Ansari et al. (2019). Increased MDA contents might be due to enhanced foliar Na^+^ concentration, which caused damage to the cell membrane. Moreover, increased proline production might be due to the disturbance of osmoregulation under high salt stress. The reason for the increased activities of antioxidants under salinity stress might be the plant’s response to alleviate salinity-induced oxidative stress (Hmaeid et al., 2019). Application of salt-tolerant rhizobacterial strains caused a significant reduction in the production of antioxidants, and the results are in line with the findings of Ansari et al. (2019) and the reason might be that the bacterial inoculants significantly decreased the detrimental impacts of NaCl on plant growth and physiology as shown by reduced Na^+^ uptake by plants and, hence, alleviated NaCl mediated metabolic disturbances in the plants (Hmaeid et al., 2019).

### Soil Enzyme Activities

Salinity stress negatively affected soil biological properties, and hence, microbially induced soil enzyme activities were disturbed (Guangming et al., 2017; Zheng et al., 2017; Raiesi and Sadeghi, 2019). This decline might be due to an indirect reduction in the soil microbial activities as well as biomass under salinity stress, which suggested the production of soil enzymes by indigenous microbes that might be lower under salinity stress. However, a tremendous increment in all the measured soil enzyme (urease, dehydrogenase, acid, and alkaline phosphatase) activities was observed in this experiment, which is in line with the findings of Hidri et al. (2019), who observed a considerable increment in the soil enzyme activities with microbially inoculated treatments. Improved dehydrogenase activities under this experiment were accompanied by a significant promotion in plant growth and nutrient profile (Rodríguez-Caballero et al., 2017).

## Conclusion

Salt-tolerant PGPR strains conferred salt tolerance to maize by facilitating the flow of essential nutrients (N, P, and K) and also suppressed the entry of Na^+^ into the plants as indicated by a decline in the K^+^/Na^+^ ratio. Reduced uptake of Na^+^ under inoculated treatments also prevented the plant from oxidative damage and membrane lipid peroxidation (MDA contents). Among all the inoculated strains, SUA-14 was proved to be the most efficient in terms of optimizing maize growth under salt stress. Improved nutrient profile under salt stress was strongly associated with the microbially mediated improvement in the soil enzyme activities, which were directly involved in the mineralization of essential nutrients and hence, modulated nutritional imbalance under salt stress. There is a need to further exploit the role of PGPR in sustaining plant growth and improving soil enzyme activities under diverse environmental conditions.

## Data Availability Statement

The datasets presented in this study can be found in online repositories. The names of the repository/repositories and accession number(s) can be found below: https://www.ncbi.nlm.nih.gov/genbank/, SUB9183410.

## Author Contributions

MS: writing—original draft preparation and investigation. MS, HA, and MFS: methodology, investigation, and formal analysis. ZZ, MFS, and HL: writing—reviewing and editing and conceptualization. HA and XZ: software, validation, visualization, and data curation. HA, HL, and XZ: supervision, project administration, and funding acquisition. All authors have contributed to the article and approved the submitted version of the manuscript.

## Conflict of Interest

The authors declare that the research was conducted in the absence of any commercial or financial relationships that could be construed as a potential conflict of interest.

## Publisher’s Note

All claims expressed in this article are solely those of the authors and do not necessarily represent those of their affiliated organizations, or those of the publisher, the editors and the reviewers. Any product that may be evaluated in this article, or claim that may be made by its manufacturer, is not guaranteed or endorsed by the publisher.
